# The safety of one-stage versus two-stage approach to osseointegrated prosthesis for limb amputation: a systematic review

**DOI:** 10.1302/2633-1462.47.BJO-2022-0117.R1

**Published:** 2023-07-21

**Authors:** Ella Banducci, Munjed Al Muderis, William Lu, Stephen R. Bested

**Affiliations:** 1 University of Notre Dame Australia, School of Medicine, Sydney, Australia; 2 St Vincent's Hospital, Sydney, Australia; 3 Department of Clinical Medicine in the Faculty of Medicine and Health Sciences, Macquarie University, Sydney, Australia; 4 Department of Orthopaedic Surgery, Macquarie University Hospital, Sydney, Australia; 5 Osseointegration International Pty Ltd, North Ryde, Australia

**Keywords:** Osseointegration, One-stage osseointegration, Two-stage osseointegration, Limb amputation, Orthopaedics, Prosthesis, osseointegrated prostheses, limb amputations, soft-tissues, bone infection, implant breakage, socket prostheses, superficial infections, infections, randomized control trial

## Abstract

**Aims:**

Safety concerns surrounding osseointegration are a significant barrier to replacing socket prosthesis as the standard of care following limb amputation. While implanted osseointegrated prostheses traditionally occur in two stages, a one-stage approach has emerged. Currently, there is no existing comparison of the outcomes of these different approaches. To address safety concerns, this study sought to determine whether a one-stage osseointegration procedure is associated with fewer adverse events than the two-staged approach.

**Methods:**

A comprehensive electronic search and quantitative data analysis from eligible studies were performed. Inclusion criteria were adults with a limb amputation managed with a one- or two-stage osseointegration procedure with follow-up reporting of complications.

**Results:**

A total of 19 studies were included: four one-stage, 14 two-stage, and one article with both one- and two-stage groups. Superficial infection was the most common complication (one-stage: 38% vs two-stage: 52%). There was a notable difference in the incidence of osteomyelitis (one-stage: nil vs two-stage: 10%) and implant failure (one-stage: 1% vs two-stage: 9%). Fracture incidence was equivocal (one-stage: 13% vs two-stage: 12%), and comparison of soft-tissue, stoma, and mechanical related complications was not possible.

**Conclusion:**

This review suggests that the one-stage approach is favourable compared to the two-stage, because the incidence of complications was slightly lower in the one-stage cohort, with a pertinent difference in the incidence of osteomyelitis and implant failure.

Cite this article: *Bone Jt Open* 2023;4(7):539–550.

## Introduction

There is significant morbidity associated with limb amputation and its prevalence is expected to increase.^1-3^ Common indications for limb amputations include trauma, tumour, infection, and peripheral vascular disease (PVD).^1,4^ The burden associated with limb amputation and increasing prevalence means that research into improved management of these patients is imperative.^1-5^

**Fig. 1:**
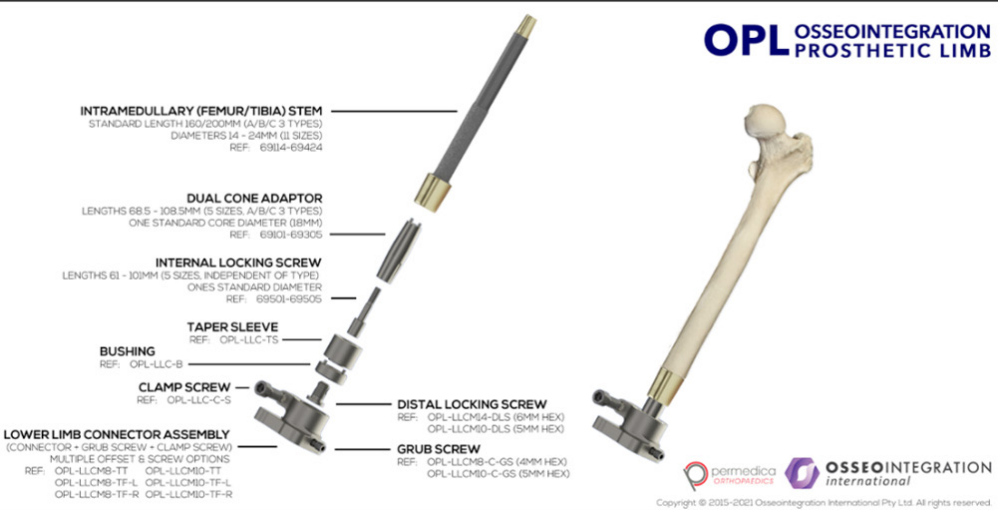
The OPL (Osseointegration Prosthetic Limb) implant: image of the prosthetic components and schematic of the implant in a femur, from Osseointegration International.

Conventionally, patients with limb amputation are treated with a socket prosthesis, where the stump sits inside the (socket of a) prosthetic device.^6^ However, patients report low satisfaction with prosthetic function and fit, or experience complications such as pain, fracture, and skin breakdown.^4,6,7^ An alternative to socket prosthesis is osseointegrated, or “bone-anchored prosthesis”.^1,4,5,8-12^ This involves direct anchorage of a prosthetic implant into residual bone via an intramedullary implant, depicted in Figure 1.

Initially described in the early 1950s by Brånemark et al,^3-5^ osseointegrated prosthesis (OIP) has become a clinically viable procedure over the last 30 years. Currently, there are multiple osseointegration systems for the treatment of amputees.^3-5,9,10,12^ Implantation of osseointegrated prosthesis traditionally occurs in two stages: implantation of the intramedullary component (S1) and the creation of a percutaneous opening for the attachment site of the prosthesis (S2).^5,13-15^ Alternatively, a one-stage approach has been developed, which involves inserting the intramedullary implant and fashioning the stoma in one procedure.^13^

However, concerns regarding the safety of osseointegration are a considerable barrier to this becoming the standard of care following limb amputation.^8,9,16^ Successful OIP relies on the host bone, the implant, and the skin-implant interface. If any of these three elements are compromised, complications can occur.^10,17^ The incidence of complications due to OIP is well reported.^8-10^ While serious adverse events, such as osteomyelitis, fracture, and implant failure, are rare, they are clinically important, as these are associated with significant morbidity.^1,8,9,16,18^

The most-reported complication of OIP is infection,^5,8-11,16,18^ and Hoellwarth et al^5^ suggested that the risk of infection is decreasing with improved management of soft-tissues. The interface between the soft-tissue and the bone-anchored implant is important in bacterial infection of the OIP due to the ‘race’ to colonize the surface of the OIP between epithelial tissue, bone, and bacteria.^5,10,17,19,20^ Since infection is unavoidable if bacteria colonize the implant prior to tissue integration,^20^ good closure of the implant-soft-tissue interface is required to prevent infectious complications.^10,17,21^ Furthermore, Hoellwarth et al^5^ noted that the risk of infection, including the risk of implant removal secondary to an infection, was reduced with the one-stage procedure because of the improved management of soft-tissues.

Interestingly, there is no literature comparing the outcomes of the one- versus two-stage approach to implantation of an OIP. Therefore, the current study contributed to the literature by determining whether there is evidence that a one-stage procedure is associated with lower infection rates compared to the two-stage approach. Furthermore, because of the significant burden associated with other osseointegration complications, such as fracture and implant failure, the scope of this review included all adverse outcomes. By identifying and comparing the incidence of adverse events after one-stage and two-stage OIPs, this study sought to determine which procedure has a favourable complication profile.

## Methods

### Search strategy

**Fig. 2:**
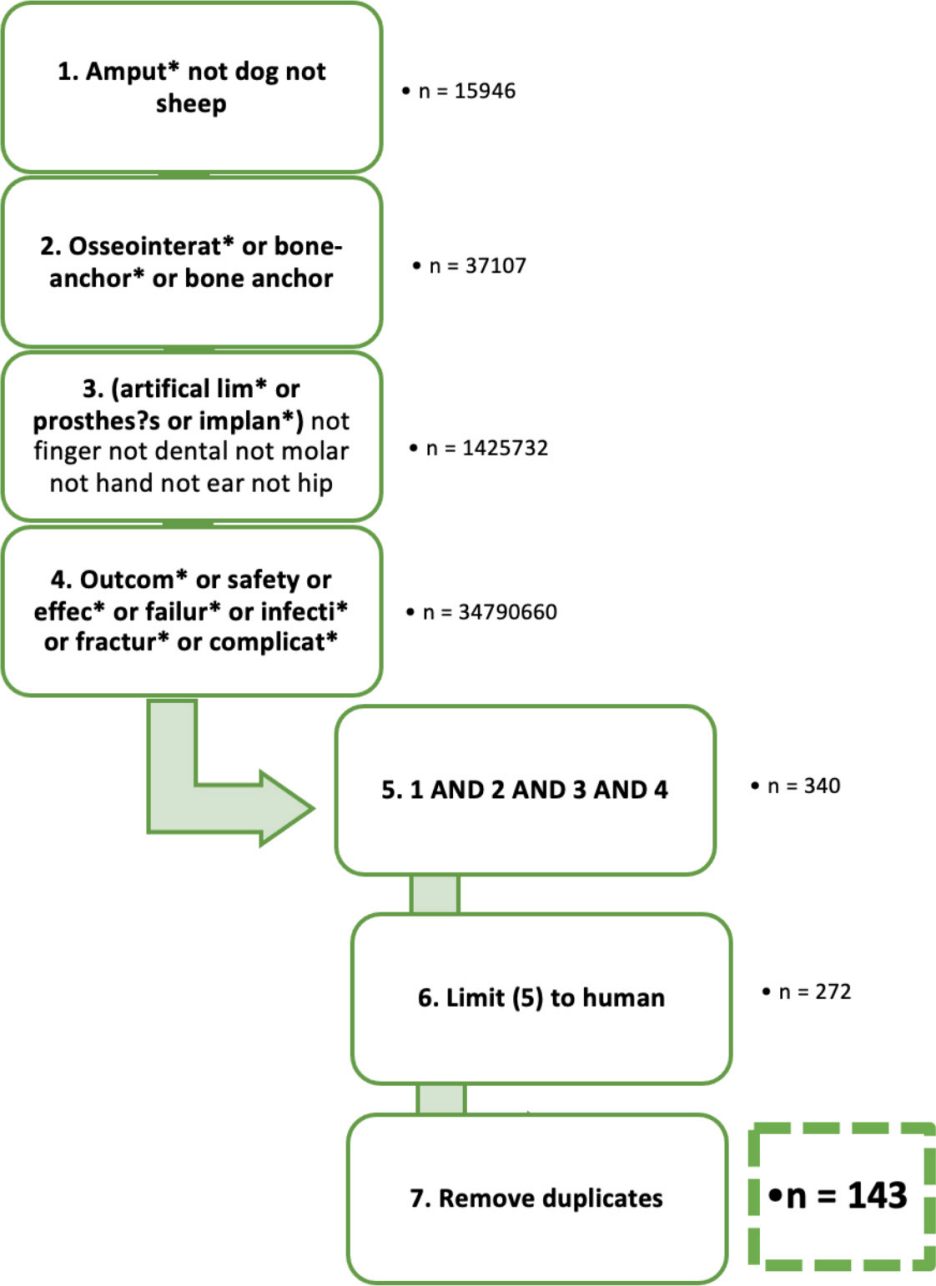
Diagrammatic representation of the electronic search strategy: keywords were based on the population (1 & 3), intervention (2), and outcome (4). Each box represents one line of the OVID advanced search string.

This systematic review followed the PRISMA guidelines.^22^ Relevant studies published before 29 December 2020 (date last searched) were identified using OVID to concurrently search MEDLINE ALL (1946 to 23 December 2020), Ovid Emcare (1995 to 2020, week 51), and Embase Classic + Embase (1947 to 24 December 2020). The electronic search strategy used a combination of MeSH and free-text keywords related to the population (e.g. amput*, artificial lim*), intervention (e.g. osseointegrat*, bone-anchor *), and outcomes (e.g. safety, failure, complicat*). The search was limited to humans, and the OVID deduplicate function was used (n = 271 to n = 143) to remove duplicated papers automatically. The full search string is provided in Figure 2. Additional relevant studies were retrieved by manually scanning the reference lists of articles identified by this search (systematic reviews) and were assessed using the same eligibility criteria.

### Eligibility criteria

The inclusion criteria were adults with an upper and/or lower limb amputation managed with a one- or two-stage osseointegration procedure, and had follow-up reporting of complications or adverse events associated with their bone-anchored prosthesis. Eligible studies were observational studies published before 29 December 2020 (i.e. date last searched).

Articles were excluded if they did not include follow-up reporting of the incidence and types of adverse events, were a conference abstract or case report, presented non-original or duplicate data, or were not in the English language.

### Study selection

**Fig. 3:**
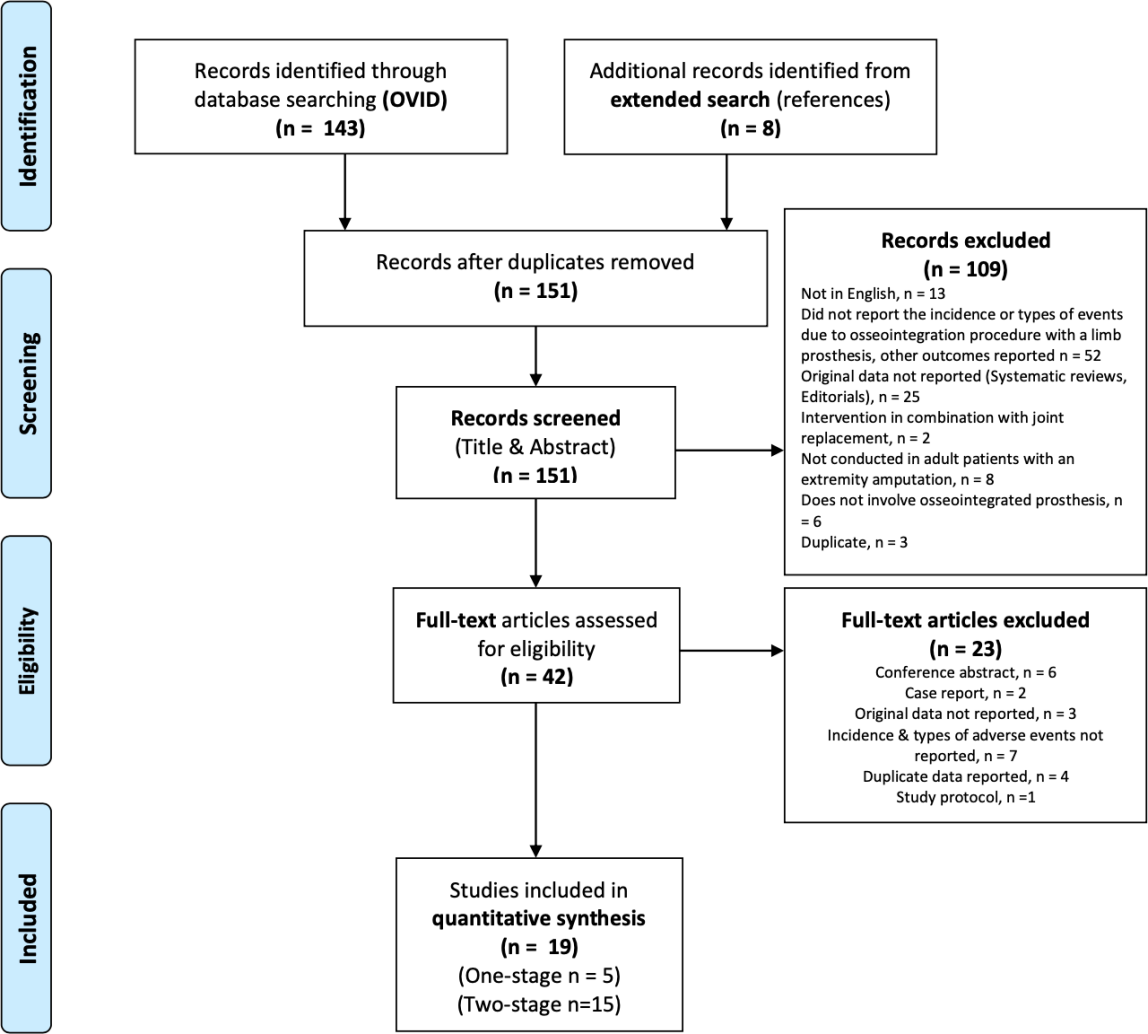
Summary of systematic review: PRISMA flow diagram depicting article selection.^22^

The electronic search results were imported into Microsoft Excel via Endnote, and study selection was then conducted in two phases. The first phase was screening the titles and abstracts to identify studies that potentially met the inclusion criteria. Additional studies were identified by manually searching the included studies’ reference lists. All studies included were evaluated in the same manner. A full-test evaluation of potentially eligible studies was conducted in phase two, and exclusion reasons were coded, as seen in Figure 3. If patient cohorts completely overlapped, the study with the most relevant data was selected.

### Data extraction

Data were extracted from eligible studies and organized into a second Microsoft Excel (USA) document, based on surgery type (i.e. one- vs two-stage). Data included were the study design, follow-up period, and all reported complications. This organization strategy revealed significant data overlap in patient cohorts, data collection periods, and follow-up duration. Because of this, quantitative pooling of data was not possible due to the extensive heterogeneity of implant design, methodology, follow-up duration, and reported complications.

### Methodological quality

The *Journal of Bone & Joint Surgery* level of evidence (LOE)^23^ rating system assesses the clinical application of research findings by study type (diagnostic, prognostic, therapeutic, economic). This hierarchical system was used to assess the study quality, with therapeutic Level I as a randomized control trial, down to Level V (mechanism-based reasoning).^23^ The Newcastle-Ottawa Scale (NOS)^24^ is a scoring system to evaluate non-randomized studies based on participant selection, comparability, and outcome determination, and was used to evaluate the risk of bias. Based on published evidence,^8^ studies scoring nine points were assessed as having a low risk of bias, seven or eight points as medium risk, and a score of six points or less was judged high-risk.

### Quantitative synthesis

Quantitative data were extracted from eligible studies, processed using Microsoft Excel to calculate mean and standard deviation. SPC for Excel was used to determine upper and lower confidence limits for outcomes of interest and generate datasets for graphs.

## Results

### Study characteristics

Table I provides a summary of study characteristics. There were 19 included studies: four reported results for the one-stage approach,^17,25-27^ 14 reported outcomes for the two-stage approach,^3,14,28-39^ and one article with both one- and two-stage groups.^40^ Osseointegration is a relatively new procedure, and included studies were published between 2010 and 2020. They were conducted across Australia, the UK, Europe (Sweden, Germany, and the Netherlands), and the USA. Included studies were Therapeutic LOE II-IV^23^ and had a high risk of bias (NOS 5 to 6).^24^

**Table I.** Study characteristics.

Author	Location	Procedure type	Implantation period, yrs	Follow-up period	Study design	Level of evidence	NOS quality score
Muderis et al^30^	Australia	Two-stage	Not recorded	Mean: 21.5 months after S1	Prospective cohort	II	5
Al Muderis et al^28^	Australia and the Netherlands	Two-stage	2009 to 2013	Median: 34 months Range: 24 to 71 months	Prospective cohort	II	5
Al Muderis^25^	Australia	One-stage	2013 to 2014	Mean: 14 months Range: 10 to 30 months	Retrospective cohort	III	5
Muderis et al^29^	Australia	Two-stage	Not recorded	Mean: 36.4 months Range: 24 to 60 months	Prospective case series	IV	5
Aschoff et al^14^	Germany	Two-stage	1999 to 2009	NR	Retrospective cohort study	III	5
Attallah et al^26^	Australia	One-stage	2015 to 2018	12 months	Multicentre case series	IV	N/A
Branemark et al^31^	Sweden	Two-stage	1999 to 2007	Two years	Prospective cohort	II	5
Branemark et al^3^	Sweden	Two-stage	1999 to 2007	Five years	Prospective cohort	II	5
Hagberg^32^	Sweden	Two-stage	1990 to 2015	Median: 7 years Range: 1 to 20 years	Retrospective cohort	III	5
Hagberg et al^33^	Sweden	Two-stage	1999 to 2017	15 years	Prospective cohort	II	5
Juhnke et al^34^	Germany	Two-stage	1999 to 2013	Range: 1 to 144 months	Retrospective comparison	III	6
Marano et al^17^	USA	One-stage	2017 to 2019	Mean: 28 weeks Range: 10 to 73 weeks	Retrospective cohort	III	5
Matthews et al^35^	UK	Two-stage	1997 to 2008	Range: 1.8 to 15.9 years	Prospective cohort	II	6
McGough et al^40^	USA	One- and Two-stage	2012 to publication (2017)	NR	Prospective cohort	II	6
Reetz et al^36^	The Netherlands	Two-stage	2009 to 2013	5 years	Retrospective cohort	III	5
Tillander et al^38^	Sweden	Two-stage	2005	Mean: 56 months Range: 3 to 132 months	Retrospective cohort	II	5
Tillander et al^37^	Sweden	Two-stage	1990 to 2010	Mean: 7.9 years Range: 1.5 to 19.6 years	Retrospective cohort	II	5
Tsikandylakis et al^39^	Sweden	Two-stage	1995 to 2010	Median: 8 years Range: 2 to 19 years	Case-series	IV	N/A
Wood et al^27^	UK	One-stage	2015 to 2017	Up to 3 years	Case-series	IV	N/A

NOS, Newcastle-Ottawa Scale; NR, not reported.

### Population characteristics

Patient characteristics are presented in Table II. Over the five papers reporting one-stage outcomes,^17,25-27,40^ the cohort size ranged from four to 22 patients (mean: 11 patients). Of the 15 studies of the two-stage approach,^3,14,28-40^ the cohort ranged from five to 111 patients (mean: 35 patients).

**Table II.** Population characteristics.

Procedure type	Author	Population		
		Number of patients	Amputation site	Indication for amputation
**One-stage**	Al Muderis et al^25^	22 patients	Unilateral TFA	Trauma, neoplasia, and infection
	Attallah et al^26^	4 patients	Unilateral TTA	Salvage knee joint alternative to above-knee amputation, excessive phantom limb pain, and socket-interface problems
	Marano et al^17^	14 patients	Lower limb - unilateral 12 × TFA 2 × TTA	Not recorded
	McGough et al^40^	6 patients	Unilateral TFA	Oncologic and traumatic
	Wood et al^27^	7 patients	6 × bilateral TFA, 1 × unilateral TFA (bilateral amputee)	Trauma (military - complex ballistic injuries)
**Two-stage**	Muderis et al^30^	50 patients	Unilateral TFA	Trauma, blast injury, infection, oncology, congenital
	Al Muderis et al^28^	86 (91 implants) 44 in Australia 42 in Norway	Unilateral TFA	Trauma, tumour, infection, congenital, other
	Muderis et al^29^	37 patients	Unilateral TFA	Not recorded
	Aschoff et al^14^	37 (39 implants)	37 × unilateral TFA, 2 × bilateral TFA	Trauma, tumour, other
	Branemark et al^31^	48 patients, 52 implants	TFA: 45 × unilateral, 6 × bilateral	Trauma, tumour, other
	Branemark et al^3^	40 patients	TFA (majority unilateral)	Trauma, tumour, other
	Hagberg^32^	12 patients	10 × bilateral TFA 2 × unilateral TFA	Not recorded
	Hagberg et al^33^	111 patients	Unilateral TFA	Trauma, tumour, emboli, infection
	Juhnke et al^34^	69 patients	65 × unilateral TFA 4 × bilateral TFA	Trauma, tumour, infection, fourth-degree burn, other
	Matthews et al^35^	18 patients	Unilateral TFA	Trauma
	McGough et al^40^	5 patients	4 × TFA, 1 × THA	Oncological, traumatic, and infection
	Reetz et al^36^	39 patients	38 × unilateral TFA 1 × bilateral TFA	Trauma, tumour, infection, other (compartment syndrome)
	Tillander et al^38^	39 patients, 45 implants	45 implants 33 × TFA, 1 × TTA, 4 × ulnar, 4 × radial, 3 × THA	Trauma or neoplasia
	Tillander et al^37^	96 patients	90 × unilateral TFA 6 × bilateral TFA	Tumour, trauma, ischaemic event, primary deep-seated infection
	Tsikandylakis et al^39^	18 patients	Unilateral THA	Trauma, tumour

TFA, transfemoral amputation; TTA, transtibial amputation.

Common indications for amputation were trauma, tumour, and infection. Patients had a variety of amputation sites: most patients had unilateral lower limb amputations (transfemoral amputation (TFA), trans-tibial amputation (TTA)), and other sites included upper limb (transhumeral amputation, bilateral, or mixed amputation sites. Common inclusion criteria were complications with socket prosthesis, skeletal maturity, ability to comply with rehabilitation protocol, and overall good health with no ongoing chemotherapy.^3,14,17,25-40^ All studies excluded patients with peripheral artery disease from receiving osseointegration surgery, except a single one-stage case series.^26^

### Complications

A tabulated summary of the incidence of complications is provided in Table III. Nine articles reported the incidence of one or more complications.^26-31,34,36,40^ Total complications of the one-stage procedure were reported in three papers,^26,27,40^ with a mean incidence of 51% (17% to 86%, SD 35%). Seven papers reported the total complications for the two-stage method,^28-31,34,36,40^ with a mean incidence of 59% (40% to 96%, SD 21%).

**Table III.** Incidence of complications.

Procedure type	Author	1 or more complication	Infection		Fracture	Soft-tissue and stoma complications		Implant failure	Mechanical complications
			Superficial	Osteomyelitis		Soft-tissue-related	Stoma-related		
**One-stage**	Al Muderis et al^25^	NR*	12/22 to 55%	Nil	Nil	6/22 to 27% elective soft-tissue refashioning		Nil	NR*
	Atallah et al^26^	2/4 to 50%	2/4 to 50%	Nil	Nil	Nil		Nil	Nil
	Marano et al^17^	NR*	2/14 to 14%	Nil	1/14 to 7%	Nil		1/14 to 7%	Not adequately reported†
	McGough et al^40^	1/6 to 17%	Nil	Nil	1/6 to 17%	Nil		Nil	Nil
	Wood et al^27^	6/7 to 86%	5/7 to 71%	Nil	3/7 to 43%	3/7 to 43% required soft-tissue refashioning		Nil	Nil
**Two-stage**	Muderis et al^30^	27 / 50 to 54%	21/50 to 42%	Nil	4/50 to 8%	10/50 to 20% required soft-tissue refashioning		2/50 to 40%	NR*
	Al Muderis et al^28^	55/86 to 64%	29/86 to 34%	Nil	3/86 to 3%	14/86 to 16% with issues related to soft-tissue	17/86 to 20% with stoma hyper granulation	3/86 to 3%	25/86 to 29%
	Muderis et al^29^	16/37 to 43%	16/37 to 43%	Nil	1/37 to 3%	6/27 to 16% elective soft-tissue refashioning		Nil	NR*
	Aschoff et al^14^	NR*	NR*	1/37 to 3%	2/37 to 5%	14/37 to 38% revision due to stoma issues		4/37 to 11%	NR*
	Brånemark et al^31^	46/48 to 96%	28/48 to 58%	4/48 to 8%	4/48 to 8%	NR*		4/48 to 8%	4/48 to 8%
	Brånemark et al^3^	NR*	34/40 to 85%	11/40 to 28%	NR*	NR*		4/40 to 10%	15/40 to 10%
	Hagberg^32^	NR*	10/12 to 83%	1/12 to 8%	2/12 to 17%	NR*		1/12 to 8%	8/12 to 67%
	Hagberg et al^33^	NR*	NR*	NR*	5/111 to 5%	NR*		18/111 to 16%	61/111 to 55%
	Juhnke et al^34^	29/69 to 42%	23/69 to 33%	1/69 to 1%	5/69 to 7%	24/69 to 35% with intervention for soft-tissue problems/ problems at stoma		4/69 to 6%	1/69 to 1%
	Matthews et al^35^	NR*	11/18 to 61%	5/18 to 28%	3/18 to 17%	NR*		5/18 to 28%	12/18 to 67%
	McGough et al^40^	2/5 to 40%	Nil	Nil	1/5 to 20%	1/5 to 20% with taper mismatch		Nil	Nil
	Reetz et al^36^	30/39 to 77%	30/39 to 77%	4/39 to 10%	NR*	14/39 to 36% required soft-tissue refashioning	8/39 to 21% with stoma hyper granulation	5/39 to 13%	Not adequately reported†
	Tillander et al^38^	NR*	NR*	7/39 to 18%	NR*	NR*		Not adequately reported†	NR*
	Tillander et al^37^	NR*	NR*	16/96 to 17%	NR*	NR*		Not adequately reported†	NR*
	Tsikandylakis et al^39^	NR*	5/18 to 28%	1/18 to 6%	8/18 to 44%	8/18 to 44% with skin irritation		3/18 to 17%	NR*

* NR = endpoint not reported.

† Not adequately reported – did not detail the number of patients with events, or total incidence of outcome (e.g. only reported septic implant failure).

### Superficial infection

Superficial infection was the most common complication, and the incidence of one or more infections was reported in 14 articles. The incidence of superficial infections from the one-stage procedure was reported in five papers,^17,25-27,40^ with a mean incidence of 38% (0% to 71%, SD 30%). The incidence of superficial infections for a two-stage approach was reported in 11 papers,^3,28-32,34-36,39,40^ and the mean incidence was 52% and ranged from 0% to 85% (SD 27%).

### Osteomyelitis (deep infection)

**Fig. 4:**
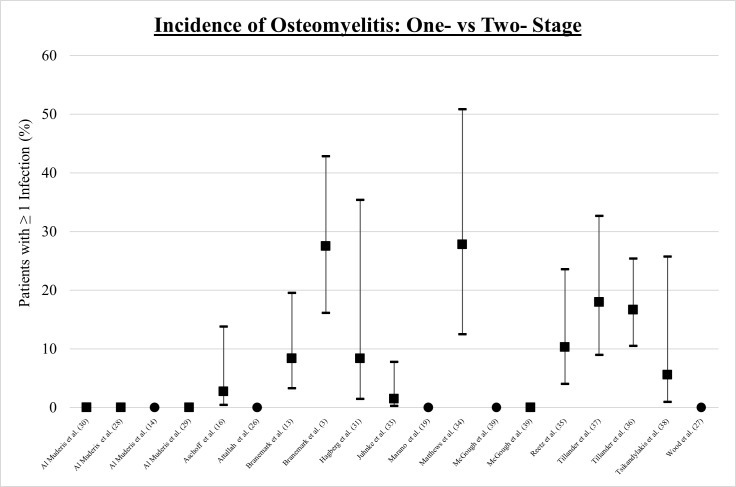
Incidence of osteomyelitis with 95% confidence intervals: ● One-stage ■ Two-stage.

Overall, 17 articles reported the incidence of osteomyelitis, depicted in Figure 4. There were no cases of osteomyelitis across five papers reporting outcomes in the one-stage cohort.^17,25-27,40^ Across the 14 articles reporting outcomes of the two-stage procedure,^3,28-32,34-36,39,40^ the mean incidence of osteomyelitis was 9%, ranging from 0% to 28% (SD 10%).

### Implant failure

**Fig. 5:**
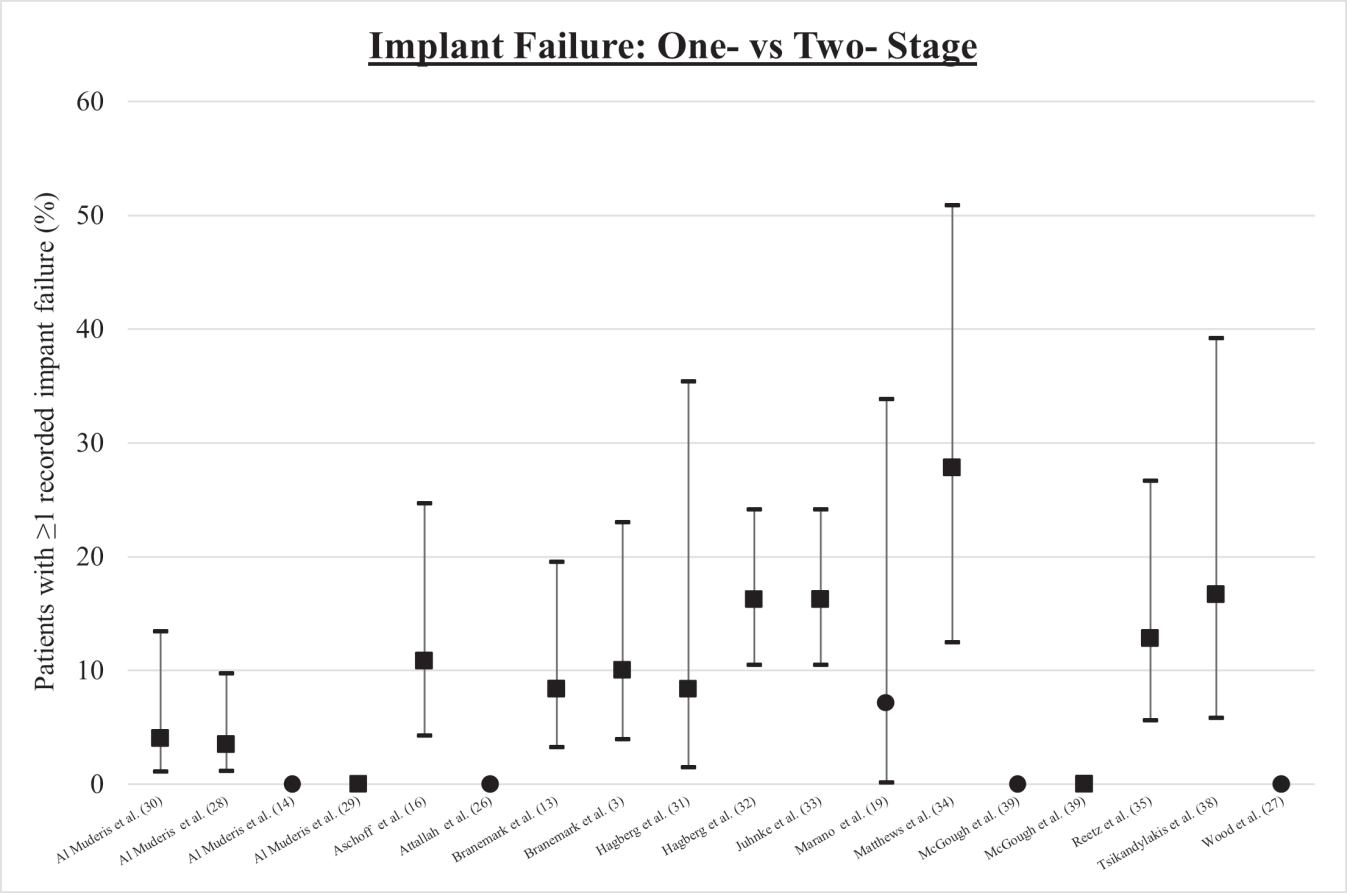
Incidence of implant failure with 95% confidence intervals: ● One-stage ■ Two-stage.

The incidence of implant failure was reported in 17 articles, depicted in Figure 5. In the five papers reporting outcomes in the one-stage cohort,^17,25-27,40^ there was one reported case of implant failure (mean 1%; SD 3%). In the 13 studies reporting outcomes of the two-stage approach,^3,14,28-36,39,40^ the mean incidence of implant failure was 9%, ranging from 0% to 28% (SD 8%).

### Fracture

Fracture incidence (one or more fractures, intraoperative or postoperative) was reported in 16 articles. The mean incidence of fractures due to the one-stage procedure was 13% (0% to 43%; SD 18%) reported in five papers (13, 18, 26, 27, 40). In the 12 articles (3, 15, 28 to 35, 39, 40) reporting fracture incidence from the two-stage approach, the mean incidence was 12% (0% to 50%; SD 14%). There was one case of intraoperative fracture in the one-stage cohort,^27^ and eight reported cases of intraoperative fracture in the first stage of the two-stage approach.^39^

### Soft-tissue/stoma-related

Soft-tissue and stoma-related complications were reported in both one- and two-stage cohorts, as seen in Table III. Reporting was infrequent and inconsistent, which prevented quantitative analysis.

### Mechanical complications

There was limited reporting of mechanical complications in both the one- and two-stage groups. Evaluation of the incidence of mechanical complications was not possible due to inconsistent reporting of the mechanism and classification of these complications.

## Discussion

Safety concerns are a considerable barrier to OIP becoming the standard of care for patients after limb amputation.^8,9,16^ Adverse events following OIP range from minor (e.g. soft-tissue infections and complications) to severe (e.g. implant infection, implant failure),^9,18,39^ and there is no literature comparing the incidence of complications of the one-stage versus two-stage approach.

Complications were common in patients treated with OIPs regardless of procedure type. In articles that reported the incidence of any complication,^26-31,34,36,40^ more than half the patients in both the one- and two-stage cohorts experienced an adverse event. Furthermore, some patients experienced more than one complication, with either several episodes of the same event, or separate complications. Thus, concerns regarding the safety of OIPs are warranted; however, socket prostheses are also associated with notable complications.^6,41^

Infection remains an important concern for patients treated with an OIP,^8,11,16^ and the primary focus of this review was to determine if there was a difference in the incidence of infection between the one- and two-stage approaches. The Al Muderis et al^28^ classification of infection related to the osseointegrated implants categorizes infection as superficial (grade 1 or 2) or deep (bone infection: grade 3, or implant failure: grade 4). This classification is important because superficial and deep infection are associated with different disease processes, treatments, and sequelae.^10,28,38^

The literature suggests that the risk of infection is decreasing with ‘improved surgical technique’ and management of the soft-tissue bone-anchored implant interface.^5,17,18^ Hoellwarth et al^5^ noted that the risk of infection was reduced with the one-stage procedure, and suggested that lower infection rates were a result of improved management of soft-tissue. Thus, because soft-tissue management is crucial to preventing infection in osseointegration procedures, ^10^ and soft-tissue optimization is the focus of the one-stage approach,^13,17^ we hypothesized that the one-stage approach enables superior soft-tissue management and subsequently results in lower infection rates.

As expected, superficial infection was the most reported complication in both the one- and two-stage cohorts.^10,18^ There was a slight difference in the incidence between the one- (37%) and two-stage (52%) approaches, favouring the one-stage procedure. This finding supports our hypothesis and suggests that the one-stage approach provides a superior soft-tissue seal, which accounts for the improved outcomes.^17,21^ However, inconsistent reporting of the number of events prohibited the comparison of event frequency between the one- and two-stage procedures.

The difference in the incidence of osteomyelitis (deep infection/grade 3 infectious complication) is the most compelling outcome of this review. Osteomyelitis is bone inflammation secondary to infection leading to bone destruction,^10,42^ and is clinically significant due to its high patient morbidity, mortality, and economic burden.^9,42^ Ideally, the surface of the OIP is colonized by bone and epithelial tissue, not bacteria,^10,17,21^ which is facilitated by tight closure of the soft-tissue bone-anchored implant interface.^5,17^ The hypothesis that the one-stage approach leads to superior stump closure, with a tight soft-tissue seal, is further supported by the fact that there were no cases of osteomyelitis in the one-stage cohort, compared to an average of 10% in the two-stage cohort. However, the risk of deep infection continues with time,^3,16,37^ and the maximum follow-up in the one-stage cohort was three years,^27^ compared to 20 years in the two-stage cohort.^32^ Furthermore, the risk of osteomyelitis may also be related to implant design.^18,37^ Overall, this review found that the one-stage procedure was associated with a lower incidence of osteomyelitis and implies that this is the more preferable approach. Because osteomyelitis is a major complication of an OIP, this finding has the potential to inform operative technique.^9,18^

Implant failure is another major complication of OIP.^9,16,18^ This is defined as implant loosening or explantation, and may be secondary to infection (septic loosening/grade 4 infection) or other processes such as failed osseointegration, implant breakage, and fatigue failure (aseptic loosening).^4,10,16^ This review found a notably lower incidence of implant failure (septic and aseptic) in the one-stage cohort, suggesting that the one-stage approach is favourable compared to the two-stage procedure. However, other factors affect osseointegration and osteoblast adhesion beyond surgical technique, such as implant design and quality of host bone.^4,10,43^

Fractures are another rare but serious adverse event associated with OIP.^9,16^ The overall definition of ‘fracture’ included periprosthetic fractures,^14,27,28,30,31,34,40^ fractures in secondary sites such as the vertebrae,^3^ fractures secondary to falls,^17,27,32,35^ and fractures secondary to septic loosening. ^35^ The average incidence of overall fractures was equivocal between the one- and two-stage approaches, as they are more likely correlated with the quality of bone and implant stability,^7,16,44^ or the patient’s return to activity.^27^ Furthermore, Hoellwarth et al^7^ suggested that the risks and complications associated with a fracture should not deter patients and clinicians, because most patients who sustained a fracture continue to wear their OIP.

Quantitative analysis and comparison of soft-tissue-, stoma-, and mechanical-related complications were not possible because of inconsistent reporting. These are areas of interest because when reported, they were not uncommon, and patients often experienced several events. Requirement for revision surgery was the most common reporting tool for soft-tissue/stoma complications;^14,25,27,29,30,34^ infrequent and inconsistent reporting of the origin (primarily stoma-related vs soft-tissue-related) prevented quantitative analysis. This is regrettable, since the evaluation of soft-tissue- and stoma-related complications between the one- and two-stage approach is an important part of assessing whether there is a difference in the quality of the soft-tissue management between these procedures. Difficulty in evaluating mechanical complications stems from inconsistent reporting (e.g. “extramedullary breakage”^28,31^ vs “dual-cone adaptor breakage”^36^ vs “mechanical complications”^15,17,33^) and the variety of osseointegrated implants used, which may have confounded the results.^4,10^

While these findings imply that the one-stage approach is preferable to the two-stage, they should be interpreted with caution, as this systematic review has several limitations. First, there was significant heterogeneity of outcome reporting, implant type, patient factors (i.e. peripheral vascular disease status, amputation site, age), rehabilitation protocols, and follow-up period, which may have confounded results. This heterogeneity and lack of clearly reported information regarding covariants, such as implant type, surgeon, and length of implant time in situ, further hinders the ability to provide robust statistical analysis to assess the optimal surgical approach. Furthermore, this limits the ability to evaluate the incidence of adverse events as a function of implant date and consider the effects of improving implant design and manufacture on the results. Second, data availability may have contributed to the distortion of the review outcomes because it was limited to published articles, with a notable overlap of patient cohorts despite efforts to prevent this in the search strategy. Third, the review was limited by the data quality because the available data had a high risk of bias, lower level of evidence (i.e. no therapeutic level I studies), generally small sample size (especially one-stage cohorts), variable follow-up periods, and inconsistent outcome reporting. Finally, the variability and low quality of the data prevented meta-analysis, limiting the analysis to a direct comparison of basic statistics.

Future research is needed to improve our understanding of the specific comparisons between one- and two-step procedures. This may include a randomized control trial that would enable control of patient and implant confounders, and a repeat review is indicated because subgroup analysis^43^ and a prospective trial^13^ of one-stage osseointegration is currently underway. Furthermore, subgroup analysis to investigate amputation site and patient factors (i.e. comorbid disease, indication for amputation), economic evaluation, and future meta-analysis of the one- versus two-stage approach are additional research areas.

The evidence analyzed by this systematic review indicates that a one-stage approach of an OIP is favourable compared to a two-stage approach. The incidence of complications was slightly lower in the one-stage cohort, especially the incidence of osteomyelitis, a clinically important complication. However, adverse events still frequently occurred in patients with OIPs treated with either approach. This review has contributed to the gap in knowledge surrounding complications and adverse events for one- and two-stage osseointegrated procedures; however, further research into soft-tissue and mechanical complications is required to appreciate the outcomes of each surgery more completely.

**Take home message**

- One-stage approach of an osseointegrated prosthesis is favourable compared to a two-stage approach.

- Incidence of complications was found to be slightly lower in the one-stage cohort.

- Of clinical importance is the reduction of osteomyelitis incidence found in the one-stage cohort when compared to the two-stage cohort.
